# Enterobacteria impair host p53 tumor suppressor activity through mRNA destabilization

**DOI:** 10.1038/s41388-022-02238-5

**Published:** 2022-02-23

**Authors:** Marie-Stéphanie Aschtgen, Konstantinos Fragkoulis, Gema Sanz, Staffan Normark, Galina Selivanova, Birgitta Henriques-Normark, Sylvain Peuget

**Affiliations:** 1 Department of Microbiology, Tumor and Cell Biology, Karolinska Institute, 171 77 Stockholm, Sweden; 2 Clinical Microbiology, Karolinska University Hospital, 171 76 Stockholm, Sweden

**Keywords:** Cancer, Microbiology

## Abstract

Increasing evidence highlights the role of bacteria in the physiopathology of cancer. However, the underlying molecular mechanisms remains poorly understood. Several cancer-associated bacteria have been shown to produce toxins which interfere with the host defense against tumorigenesis. Here, we show that lipopolysaccharides from *Klebsiella pneumoniae* and other Enterobacteria strongly inhibit the host tumor suppressor p53 pathway through a novel mechanism of p53 regulation. We found that lipopolysaccharides destabilize *TP53* mRNA through a TLR4-NF-κB-mediated inhibition of the RNA-binding factor Wig-1. Importantly, we show that *K. pneumoniae* disables two major tumor barriers, oncogene-induced DNA damage signaling and senescence, by impairing p53 transcriptional activity upon DNA damage and oncogenic stress. Furthermore, we found an inverse correlation between the levels of TLR4 and p53 mutation in colorectal tumors. Hence, our data suggest that the repression of p53 by Enterobacteria via TLR4 alleviates the selection pressure for p53 oncogenic mutations and shapes the genomic evolution of cancer.

Over the last decades, the human microbiome has emerged as an important regulator of the physiopathology of cancer and it has been estimated that infections could be the main driver for over 20% of cancers [1]. Recent evidence highlights the role of the bacterial microbiota in cancer initiation, progression and response to therapy [2]. Enrichment of specific bacterial species has been found in cancer patients by metagenomics study, linking bacterial dysbiosis to cancer [3, 4]. Moreover, the ability for oncogenic bacteria to act as cancer drivers and to initiate tumorigenesis has been demonstrated for *Helicobacter pylori* [5] as well as for genotoxin-producing bacteria [6, 7]. Several other bacterial species have also been experimentally associated to increased cancer risk, such as *Fusobacterium nucleatum* [8]. Overall, the essential role of the bacterial microbiota in cancer is now well established. However, despite recent advances, the underlying molecular mechanisms by which our microbiota influences cancer remain elusive.

Bacteria produce multiple toxins, metabolites and pro-inflammatory molecules which directly or indirectly target host signaling pathways involved in all the cancer hallmarks and thereby interfere with the host defense against tumorigenesis, such as the p53 pathway. The tumor suppressor p53, encoded by the *TP53* gene, is a transcription factor which acts as a cell signaling hub to integrate various cell stresses into an appropriate cell response [9]. Upon stress, phosphorylation events lead to the dissociation of p53 from its ubiquitin-ligase MDM2, preventing its degradation by the proteasome and leading to its accumulation in the nucleus. p53 controls transcription programs regulating multiple cell functions including apoptosis, cell cycle arrest and DNA damage response, and acts as the main barrier against tumorigenesis. Additionally, p53 has been long known to be involved in host defense against viral infection, due to its role in the DNA damage response, and hence it is specifically targeted by oncogenic viruses [10, 11]. Interestingly, known oncogenic bacteria such as *H. pylori* [12–14], *Chlamydia trachomatis* [15] and several *Mycoplasma* species [16] also interfere with the p53 pathway. Altogether, these studies suggest that p53 inactivation could be a more general hallmark of microbes with tumorigenic potential.

*Klebsiella pneumoniae* is an opportunistic pathogen of the digestive and upper respiratory tract. Metagenomics and clinical epidemiologic data suggest that *K. pneumoniae* could play a role in cancer initiation and progression*. K. pneumoniae* has been shown to be enriched in the gut of colorectal cancer patients (CRC) [17]. Moreover, hypervirulent strains of *K. pneumoniae*, which cause pyogenic liver abscesses, have been associated with increased risk of CRC [18, 19]. Interestingly, several hypervirulent *K. pneumoniae* strains harbor the *pks* locus encoding for the genotoxin colibactin which could be involved in *K. pneumoniae* tumorigenic potential [20]. However, the mechanisms that link *K. pneumoniae* and cancers are still poorly understood. In this study, we investigate the impact of *K. pneumoniae* on the host p53 pathway. We find that lipopolysaccharide (LPS) from *K. pneumoniae* and other Enterobacteria inhibits p53 through the TLR4-NF-κB pathway, and we uncover a novel mechanism of p53 regulation whereby p53 inhibition occurs at the mRNA level.

## Results

### Klebsiella pneumoniae inhibits p53

**Fig. 1:**
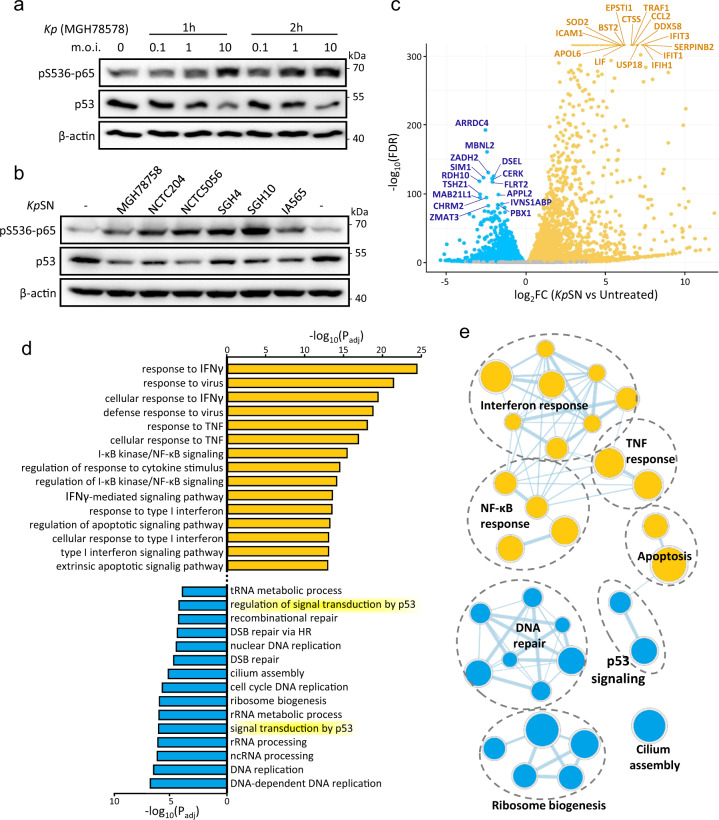
*Klebsiella pneumoniae* inhibits p53. **a** NF-κB activation (followed by p65 phosphorylation) and p53 protein level in BJ hTert cells upon infection by the *K. pneumoniae* (*Kp*) strain MGH78758 at different m.o.i. **b** Western blot of BJ hTert cells upon exposure to supernatant from *K. pneumoniae* culture (*Kp*SN), from different *K. pneumoniae* strains. **c** RNA-seq data of BJ hTert cells exposed to *Kp*SN, highlighting top15 upregulated (orange) and downregulated genes (blue). **d** GO pathway enrichment analysis of the top up- and downregulated genes upon *Kp*SN. **e**, Network visualization of the top up- and downregulated pathways.

To investigate the effect of *K. pneumoniae* on the host p53 pathway, we infected human immortalized fibroblasts (BJ hTERT) with live bacteria. We observed a robust downregulation of p53 protein level, concomitant with the activation of NF-κB, as demonstrated by Ser536 phosphorylation of the NF-κB subunit p65 (RelA) (Fig. 1a). Interestingly, p53 downregulation was bacteria-cell contact independent and was recapitulated when treating the cells with supernatant from *K. pneumoniae* culture (*Kp*SN), independently of the *K. pneumoniae* strains used (Fig. 1b and Supplementary Fig. S1a-b). Pathway analysis of gene expression changes in human fibroblasts exposed to *Kp*SN, measured by RNA-seq, highlighted a robust activation of the canonical immune and inflammatory pathways involved in the host response to bacteria, as expected (Fig. 1c-d). Notably, the analysis of genes downregulated upon *Kp*SN revealed a significant inhibition of the p53 signaling pathway and DNA damage response (Fig. 1d). These data suggest that *K. pneumoniae*-mediated p53 protein decline is sufficient to impair the biological function of p53.

### *Klebsiella pneumoniae* impairs the p53 response to DNA damage and oncogenic stress

**Fig. 2:**
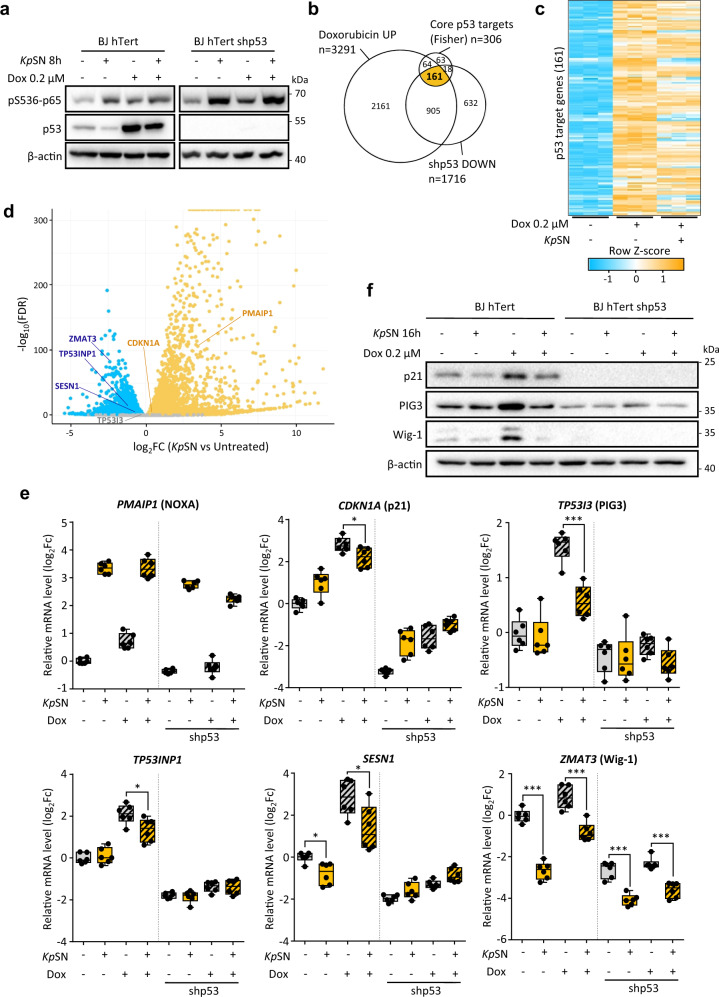
*Klebsiella pneumoniae* impairs p53 response to DNA damage. **a** Western blot of BJ hTert cells treated with the DNA-damaging agent doxorubicin (Dox) and with *Kp*SN. **b** High confidence set of p53 target genes obtained by intersecting the core p53 target gene set define by Fisher et al. with genes upregulated by doxorubicin and downregulated upon p53 knock-down in our RNA-seq data from BJ hTert cells. **c** RNA-seq data of high-confidence p53 target genes expression upon doxorubicin and *Kp*SN. **d** Expression change of selected well-characterized p53 target genes in RNA-seq data of BJ hTert cells exposed to *Kp*SN. **e** RT-qPCR validation of selected p53 target genes in BJ hTert cells upon exposure to doxorubicin, *Kp*SN and p53 knockdown. **f** Western blots of BJ hTert cells (wt and p53 knockdown) showing protein level of p53 target genes upon 16 h exposure to doxorubicin and *Kp*SN. *p < 0.05; ***p < 0.01.

To confirm that *K. pneumoniae*-induced downregulation of p53 impacts its transcriptional activity, we investigated the effect of *Kp*SN on p53 level and activity upon doxorubicin-induced DNA damage, which is well known to stabilize p53 and activate its target genes. First, we confirmed that upon DNA damage, the stabilization of p53 is impaired in *Kp*SN treated cells (Fig. 2a). Then, we investigated how it affects p53-dependent transcription. The set of genes regulated by p53 is highly dependent on the cell context [21]. Therefore, we defined a gene set of 161 high confidence p53 targets in our model. We intersected genes upregulated upon DNA damage in a p53-dependent manner in BJ-hTERT fibroblasts (which were not induced by doxorubicin upon p53 shRNA according to our RNA-seq data) with the p53 core targets gene set defined by Fisher et al. [22] (Fig. 2b). Then, we compared how *Kp*SN affected doxorubicin-induced activation of our gene set of 161 high confidence p53 targets. Our analysis revealed an overall inhibition of p53 targets by *Kp*SN upon DNA damage (Fig. 2c). Using RT-qPCR, we investigated the expression of well characterized p53 target genes *PMAIP1* (NOXA), *CDKN1A (*p21), *TP53I3* (PIG3), *SESN1*, as well as *ZMAT3* (Wig-1) and *TP53INP1* which have been recently highlighted as major effectors of p53-mediated tumor suppression [23, 24] (Fig. 2d-e). Interestingly, both *PMAIP1* and *CDKN1A* were upregulated by *Kp*SN in a p53-independent manner. However, *Kp*SN partially prevented a robust p53-mediated induction of *CDKN1A* upon doxorubicin and strongly impaired the induction of *TP53I3*, *SESN1* and *TP53INP1*. Notably, while we observed a diminished *ZMAT3* induction upon DNA damage, *ZMAT3* was repressed by *Kp*SN in a p53-independent manner (Fig. 2e). We then confirmed the *Kp*SN-induced impairment of the p53 response to DNA damage at protein level (Fig. 2f).

**Fig. 3:**
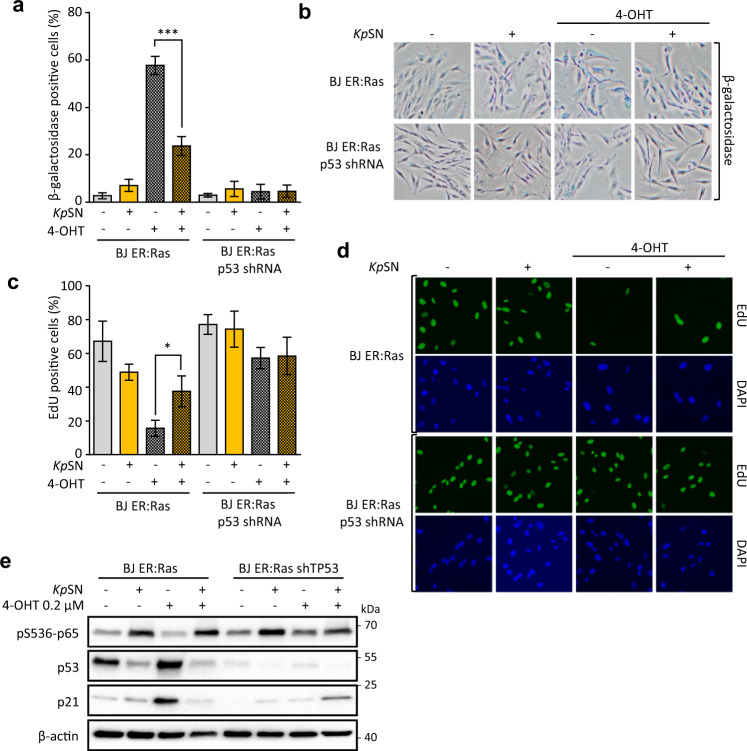
*Klebsiella pneumoniae* impairs p53 response to oncogenic stress. Oncogenic Hras was activated for 7 days by 4-OHT in BJ hTert HRasV12^ER-Tam^ inducible fibroblasts to cause replication stress. **a** Senescence induced by Hras activation was monitored by quantification of β-galactosidase positive cells upon exposure to *Kp*SN or p53 knock-down. **b** Representative images of β-galactosidase staining. **c** Senescence-associated replication arrest was assessed by microscopy-based EdU incorporation assay. **d** Representative fluorescence microscopy images of EdU Staining. DAPI was used to counterstain nuclei. **e** Protein levels of phospho-p65, p53 and p21 after 72 h of RAS induction. *p < 0.05; ***p < 0.01.

To investigate the biological consequences of *K. pneumoniae*-induced inhibition of p53, we used BJ fibroblasts with an inducible oncogenic Ras system (BJ hTert HRasV12^ER-Tam^) as a model of oncogenic stress. In these cells, activation of oncogenic Hras by 4-hydroxytamoxifen (4-OHT) induces a strong p53- and p21-dependent senescence [25]. We monitored senescence by increase in β-galactosidase activity (Fig. 3a-b) and by cell replication arrest revealed by EdU incorporation assay (Fig. 3c-d). We found that *Kp*SN impairs p53-dependent induction of senescence upon oncogene activation, as well as prevents accumulation of p53 and p21 (Fig. 3e).

Overall, our data show that secreted factors from *K. pneumoniae* downregulate p53 protein level upon DNA damage and oncogenic stress, which impairs its transcriptional activity and its tumor suppressive function.

### *Klebsiella pneumoniae* lipopolysaccharide (LPS) is sufficient to inhibit p53

**Fig. 4:**
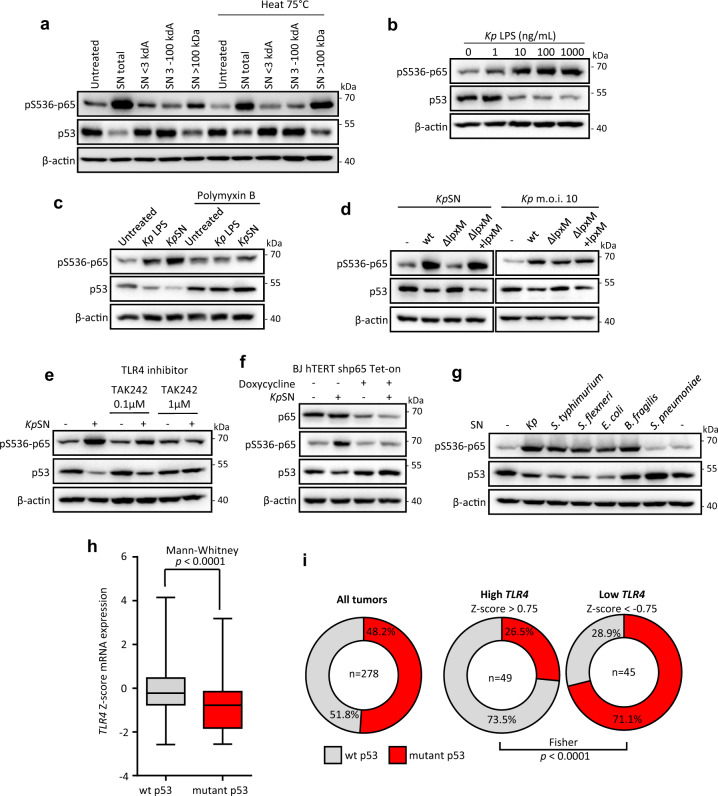
*Klebsiella pneumoniae* LPS is sufficient to inhibit p53. **a** Western Blot of BJ hTert cells treated with different *Kp*SN fractions. **b** Western blot of BJ hTert cells upon exposure to purified *K. pneumoniae* LPS. **c** Effect of LPS inactivation by polymyxin B on BJ hTert cells response to LPS or *Kp*SN assessed by Western blot. Culture medium containing purified LPS (100 ng/mL) or *Kp*SN was treated 24 h with 30 μg/mL of polymyxin B sulfate before addition to cells. **d** Western blot of BJ hTert cells infected by *K. pneumoniae Δlpxm* or exposed to *Kp*SN from *Δlpxm* bacteria. *lpxm* deletion was complemented using a pBAD plasmid expressing lpxM (*Δlpxm* + *lpxm)*. **e** TLR4 dependency of BJ hTert cells response to *Kp*SN assessed by Western blot upon treatment with TLR4 inhibitor TAK242. **f** NF-κB dependency of BJ hTert cells response to *Kp*SN assessed by Western blot using Tet-inducible p65 knock-down BJ hTert cells. p65 shRNA was induced by doxycycline for 48 h before the experiment. **g** Protein level of phospho-p65 and p53 in BJ hTert cells upon exposure to culture supernatant from different bacteria species. **h** Negative correlation between TLR4 expression and p53 mutation in early grade colorectal adenocarcinoma patients data (COAD, TCGA) suggests that TLR4-driven inhibition of p53 alleviate the selection pressure for p53 mutation in cancer. **i** Comparison of p53 mutation rate between early colorectal adenocarcinoma with low and high TLR4 expression.

We then investigated which *K. pneumoniae* secreted factor is responsible for host p53 downregulation. We performed a molecular weight fractionation of *Kp*SN and heat treatment (Fig. 4a). The effect on p53 was recapitulated by the heat-resistant fraction of high molecular weight (>100 kDa). This fraction contains bacterial outer membrane vesicles (OMVs) associated with LPS, suggesting that LPS could be the signal for p53 downregulation. Indeed, purified LPS from *K. pneumoniae* induced a robust downregulation of p53 protein in immortalized fibroblasts in a dose-dependent manner (Fig. 4b). We confirmed this effect in primary human fibroblasts, M1 and M2 monocytes-derived macrophages, and melanoma cell line A375 (Supplementary Fig. S2a). Moreover, addition of polymyxin B to the culture medium, known to block the effects of LPS through binding to lipid A, completely rescued the downregulation of host p53 (Fig. 4c). To confirm the involvement of lipid A, we generated a *K. pneumoniae* deletion mutant for the *lpxM* gene, which encodes a myristoyl transferase that catalyzes the final step of lipid A biosynthesis (Supplementary Fig. S2b). Deletion of *lpxM* did not affect bacterial growth nor the amount of LPS secreted (Supplementary Fig. S2c, d). However, *K. pneumoniae* Δ*lpxM* was unable to affect p53 protein level in host cells, neither upon direct infection nor upon treatment with bacterial supernatant, while the complemented strain restored the downregulation (Fig. 4d). Altogether, these results demonstrate that *K. pneumoniae* inhibits p53 through the lipid A of its LPS.

To further examine which host pathway is involved, we performed similar experiments while inhibiting the classical LPS signaling pathway, including its receptor TLR4 or the major cellular mediator NF-κB. Both TLR4 inhibitor and Tet-inducible knockdown of the NF-κB subunit p65 completely rescued the inhibition of host p53 (Fig. 4e, f). As the TLR4-NF-κB axis is a canonical signal transduction pathway activated by LPS from most Gram-negative bacteria, we investigated if the effect on p53 is shared with other bacterial species (Fig. 4g and Supplementary Fig. S2e). Indeed, culture supernatants from other Enterobacteria (*Salmonella typhimurium*, *Shigella flexneri*, *Escherichia coli*) also strongly repressed p53. In contrast, p53 was activated by Gram-positive *Streptococcus pneumoniae*. Importantly, we did not observe p53 repression by LPS from commensal Gram-negative *Bacteroides fragilis*, which is known to have a penta-acetylated lipid A structure acting as TLR4 antagonist [26]. However, p65 was phosphorylated to a similar extent, suggesting that besides the canonical NF-κB pathway, other transduction pathways are involved in p53 repression.

Next, we explored if the inhibition of p53 by LPS/TLR4 could play a role in tumor initiation or progression, using data from cancer patients deposited in the Cancer Genome Atlas (TCGA). We hypothesized that inhibition of p53 by bacterial signaling could alleviate the selection pressure for p53 mutation in *TLR4* expressing tumors. Hence, we investigated the relationship between the *TLR4* expression level and the p53 mutation rate in early-stage colorectal adenocarcinoma (grade I-II). We observed a robust correlation (*p* < 0.0001) between *TLR4* mRNA level and absence of a p53 mutation in the tumors (Fig. 4h). Moreover, comparison between tumors with high TLR4 (Z-score > 0.75) and low TLR4 (Z-score < −0.75) revealed a striking inversion of the p53 mutation rate (Fig. 4i). While these data could be explained by a transcriptional control of TLR4 by wild-type p53, as previously suggested by Menendez *et al*. [27], we did not observe the same correlation in other tumor contexts known to have no or low bacterial load (such as breast and liver cancers) or with a lower abundance of Gram-negative bacteria (such as lung cancer) [28] (Supplementary Fig. S3a, b). These data suggest that activation of the TLR4-NF-κB by LPS from the microbiota could contribute to tumorigenesis by restricting p53 tumor suppressive function and shed a new light on the known pro-tumorigenic effect of LPS [29].

### p53 inhibition occurs at the mRNA level

**Fig. 5:**
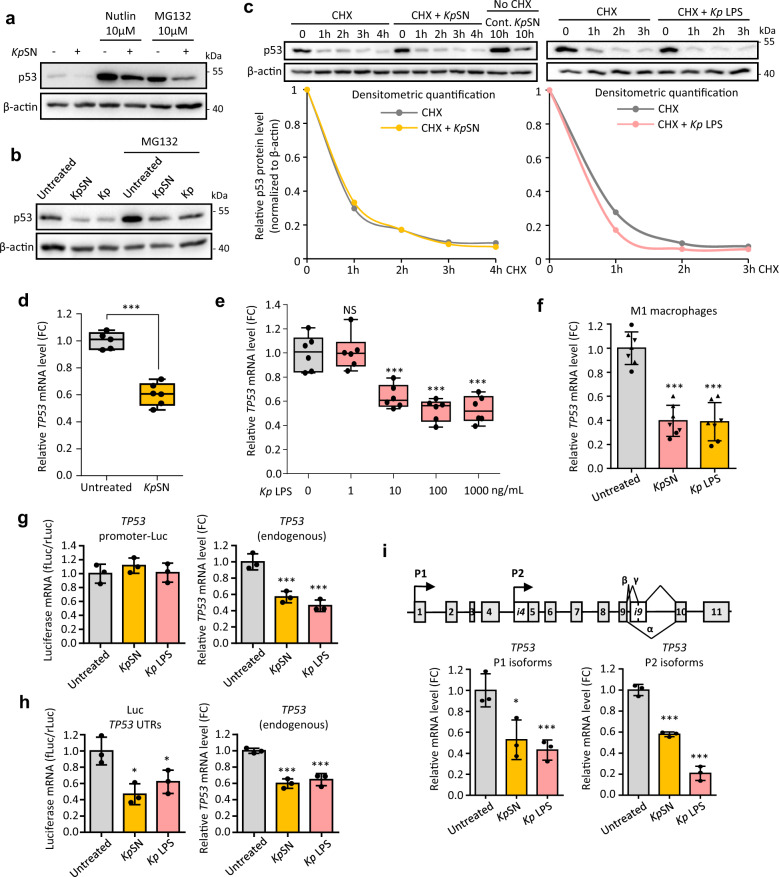
p53 inhibition occurs at mRNA level. **a** Downregulation of p53 protein level induced by *Kp*SN in BJ hTert cells is not rescued by MDM2 inhibitor Nutlin or by proteasome inhibitor MG132. **b** Proteasome inhibition by MG132 does not rescue p53 downregulation upon direct infection of BJ hTert cells by *K. pneumoniae* (m.o.i. 10). **c** p53 protein stability in BJ hTert cells exposed to *Kp*SN (orange) or 100 ng/mL LPS (pink) was investigated using cycloheximide (CHX) chase assay. Cells were pretreated 4 h with *Kp*SN before CHX treatment. Lower panels show the densitometry quantification of Western blots bands normalized to β-actin level. **d** RT-qPCR for *TP53* mRNA level of BJ hTert cells upon 8 h exposure to *Kp*SN, assessed by. **e**, RT-qPCR for *TP53* mRNA level of BJ hTert cells upon 8 h exposure to indicated dose of LPS. **f** RT-qPCR for *TP53* mRNA level upon *Kp*SN or 100 ng/mL LPS (8 h) in PBMC-derived macrophages, polarized into M1 phenotype. The different data point symbols indicate different blood donors. **g** Luciferase reporter assay using *TP53* promoter-Luc construct transfected in BJ hTert cells. Transfection rate was normalized by co-transfection of *Renilla* luciferase vector. Endogenous *TP53* mRNA level was measured in the same samples. **h** Luciferase reporter assay using a construct expressing the luciferase flanked by p53 UTRs, transfected in BJ hTert cells. Transfection rate was normalized by co-transfection of *Renilla* luciferase vector. Endogenous *TP53* mRNA level was measured in the same samples. **i** Upper panel: structure of the *TP53* gene with the two alternative promoters P1 and P2. Lower panel: mRNA level of P1 and P2 p53 isoforms in BJ hTert cells upon treatment with *Kp*SN or 100 ng/mL LPS (8 h). *p < 0.05; ***p < 0.01; NS non-significant.

p53 has been extensively described to be regulated solely at the protein level, through modification of its stability, conformation and/or protein interactions [9]. To ensure a fast response to stress, p53 is constantly produced and targeted to proteasomal degradation through MDM2-dependent polyubiquitination. Several bacterial toxins have been described to enhance p53 degradation through AKT-mediated activation of MDM2 [30]. Calpain-cleavage of p53 by bacteria has also been reported [31]. Surprisingly, we found that downregulation of p53 by *Kp*SN did not involve enhanced proteasomal degradation. While p53 was indeed stabilized upon proteasome inhibition by MG132 or MDM2 inhibition by Nutlin, we did not observe any prevention of the p53 decline induced by *Kp*SN or by *K. pneumoniae* direct infections (Fig. 5a-b). Similarly, doxorubicin-induced DNA damage (which leads to disruption of the p53-MDM2 complex and p53 stabilization) did not rescue *Kp*SN-induced p53 downregulation (Fig. 2a). Inhibition of AKT or calpain also both failed to rescue p53 protein level (Supplementary Fig. S4a). Moreover, analysis of p53 protein stability by cycloheximide chase assay did not reveal any significant change in p53 stability when cells were exposed to *Kp*SN or LPS (Fig. 5c).

Since our data showed that p53 protein stability was not affected, we investigated the *TP*53 mRNA by RT-qPCR and found that *Kp*SN strongly decreased *TP*53 mRNA level in human immortalized fibroblasts (BJ hTert) (Fig. 5d-e), normal primary fibroblasts (HNF) and in A375 melanoma cells (Supplementary Fig. S4b-c). This effect was recapitulated in a dose-dependent manner upon treatment of cells with *K. pneumoniae* LPS (Fig. 5e and Supplementary Fig. S4d). We also observed the repression of *TP*53 mRNA level in M1 and M2 monocytes-derived macrophages upon treatment with *Kp*SN or LPS (Fig. 5f and Supplementary Fig. S4e). Finally, inhibition of TLR4 or deletion of *K. pneumoniae lpxM* prevented *TP53* mRNA downregulation (Supplementary Fig. S4f, g).

*TP53* gene transcription has previously been described to be repressed by phosphorylated STAT3 upon LPS [32]. However, we did not observe a rescue of *TP*53 mRNA in *STAT3*-depleted cells (Supplementary Fig. S4h-i). Taken together, our results suggest a novel mechanism of p53 regulation by LPS-TLR4-NF-κB through STAT3-independent regulation of *TP*53 mRNA.

To identify the mechanism of *TP53* mRNA inhibition by *K. pneumoniae*, we tested whether *TP53* mRNA expression was affected at the promoter level or post-transcriptionally through *TP53* mRNA UTRs. We performed dual luciferase reporter assays using a construct where the luciferase expression is under the control of the *TP53* promoter (*TP53* promoter-Luc) and another construct constitutively expressing the luciferase mRNA flanked by TP53 untranslated regions (Luc-*TP53* UTRs). Inhibition of endogenous *TP53* was controlled in the same sample by RT-qPCR. We did not observe any change in luciferase expression of *TP53* promoter-Luc upon treatment with *Kp*SN or LPS (Fig. 5g), suggesting that the promoter activity of *TP*53 is not affected. However, both *Kp*SN and LPS induced a robust decrease in luciferase from the Luc *TP53* UTRs plasmid (Fig. 5h), indicating that *Kp*SN and LPS might regulate *TP*53 mRNA stability through its UTRs. To find out which UTR was involved, we investigated the regulation of different p53 isoforms (Fig. 5i). *TP*53 gene encodes two families of p53 isoforms through an alternative promoter P2 located in intron 4, which regulates the expression of short isoforms (Δ133 and Δ160 p53 isoforms), while the full length isoforms are regulated by its main promoter P1 [33]. P1 and P2 isoforms share their 3ʹ-UTR but have different 5ʹ-UTR sequence and different promoter regulation. Using RT-qPCR with isoform-specific primers, we found that both isoform families are similarly repressed by *Kp*SN and LPS, suggesting a regulation of the *TP53* 3ʹ-UTR (Fig. 5i).

### LPS-induced downregulation of *ZMAT3* destabilizes *TP53* mRNA

**Fig. 6:**
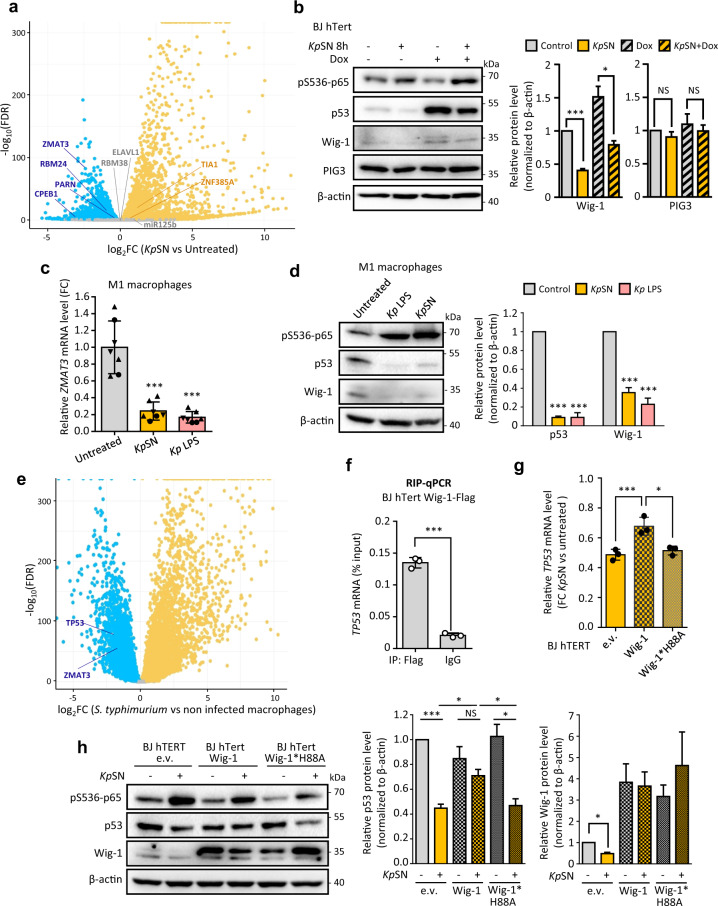
LPS-induced downregulation of *ZMAT3* destabilizes *TP53* mRNA. **a** Differential expression level of known factors regulating TP53 3ʹUTR in our RNA-seq data of *Kp*SN-treated BJ hTert cells. **b** Left panel: Western blot of BJ hTert cells upon 8 h treatment with *Kp*SN and doxorubicin. Right panel: densitometric quantification of Wig-1 and PIG3 Western blot bands normalized to β-actin level. **c** RT-qPCR for *ZMAT3* mRNA level upon *Kp*SN or 100 ng/mL LPS (8 h) in PBMC-derived macrophages, polarized into M1 phenotype. The different data point symbols indicate different blood donors. **d** Left panel: Western blot of M1 macrophages upon *Kp*SN or LPS treatment. Right panel: densitometric quantification of p53 and Wig-1 Western blot bands normalized to β-actin level. **e** mRNA level of *TP53* and *ZMAT3* in RNA-seq data from PBMC-derived macrophages from 60 different blood donors infected by *Salmonella thyphimurium*. **f** Validation of Wig-1 binding to *TP53* mRNA in BJ hTert cells by RIP-qPCR. **g** Rescue of *Kp*SN-induced p53 downregulation in BJ hTert cells by Wig-1 overexpression but not by the RNA-binding deficient H88A mutant, assessed by RT-qPCR. **h** Left panel: Western blot of Wig-1 and Wig-1*H88A overexpressing BJ hTert cells upon *Kp*SN treatment. Right panels: densitometric quantification of p53 and Wig-1 Western blot bands normalized to β-actin level. *p < 0.05; ***p < 0.01; NS non-significant.

We then investigated the expression of genes known to regulate *TP53* mRNA by binding its 3ʹUTR (Fig. 6a) [34]. Interestingly, we observed that several genes encoding factors stabilizing *TP53* mRNA (such as *ZMAT3* or *CPEB1*) [35, 36] were repressed upon exposure to *Kp*SN, while factors known to inhibit its translation (such as *Tia1*) [37] where activated. Strikingly, *ZMAT3*, which encodes the RNA binding protein Wig-1, was one of the top repressed genes in our RNA-seq data (Fig. 1c). Wig-1 has been shown to bind to an A/U-rich element (ARE) on *TP53* 3ʹUTR to stabilize *TP53* mRNA [35]. Moreover, *ZMAT3* forms a regulatory feedback loop with p53 since it is a p53 target gene crucial for p53 tumor suppression [23, 24]. As we already showed that *ZMAT3* repression upon *Kp*SN is p53 independent (Fig. 2e**)**, we tested if Wig-1 was inhibited at earlier time points (8 h) than other p53 target genes. We observed the downregulation of both isoforms of Wig-1 at the same time frame as *TP53* mRNA but before other p53 targets such as PIG3 (Fig. 6b and Fig. 2f). Moreover, Wig-1 was repressed at mRNA and protein level in M1 and M2 monocytes-derived macrophages (Fig. 6c, d and Supplementary Fig. S5a-b). Analysis of RNA-seq data from PBMC-derived macrophages from 60 different blood donors infected by *Salmonella thyphimurium* [38] confirmed the robust inhibition of both *TP53* and *ZMAT3* upon direct infection of macrophages by another Enterobacterial species (Fig. 6e).

**Fig. 7:**
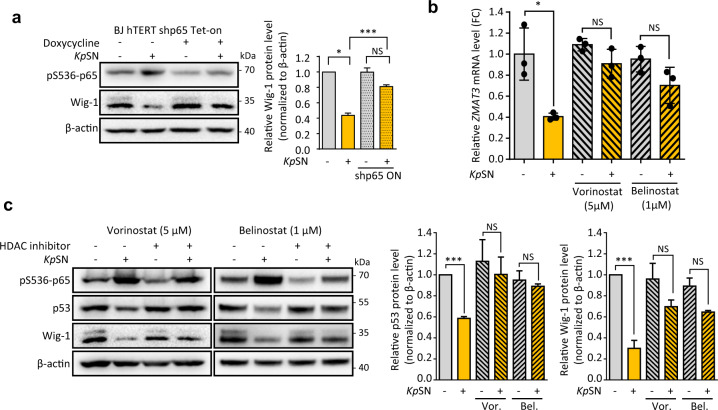
LPS-induced downregulation of *ZMAT3* is NF-κB and HDAC-dependent. **a** NF-κB dependency of Wig-1 downregulation upon *Kp*SN assessed by Western blot using Tet-inducible p65 knock-down BJ hTert cells. p65 shRNA was induced by doxycycline for 48 h before the experiment. Right panel: densitometric quantification of Wig-1 Western blots bands normalized to β-actin level. **b** RT-qpCR for *Kp*SN-induced differential expression of *ZMAT3* in BJ hTert cells upon HDAC inhibition by pan-HDAC inhibitors vorinostat and belinostat. **c** Left panel: Western blot of BJ hTert cells exposed to *Kp*SN upon HDAC inhibition. Right panels: densitometric quantification of p53 and Wig-1 Western blot bands normalized to β-actin level. *p < 0.05; ***p < 0.01; NS non-significant.

To confirm the role of Wig-1 in p53 downregulation, we validated the binding of Wig-1 to *TP53* mRNA by RIP-qPCR in our model (Fig. 6f). Then, we established fibroblasts stably overexpressing wild type Wig-1 (BJ hTert pLV Wig-1) or a zinc-finger mutant unable to bind *TP53* mRNA [35] (BJ hTert pLV Wig-1*H88A). Overexpression of wt Wig-1, but not of the H88A mutant, partially rescued both *TP53* mRNA and p53 protein levels (Fig. 6g-h), demonstrating that repression of *ZMAT3* gene by *Kp*SN participates in the destabilization of *TP53* mRNA. Finally, *ZMAT3* repression also affected the stability of several others mRNA, previously described to be either stabilized (such as *EIF4B* and *RRM1*) [39] or destabilized by Wig-1 (such as *FAS*) [40], suggesting a broader effect than p53 inhibition (Supplementary Fig. S5c, d). We then investigated the mechanism of *ZMAT3* repression. We found that knock-down of p65 rescued Wig-1 downregulation, confirming the NF-κB involvement in *ZMAT3* repression by *Kp*SN (Fig. 7a). LPS-induced repression of genes through NF-κB has been recently shown to be regulated by histone deacetylases (HDACs) [41]. Using two different pan-HDACs inhibitors, vorinostat and belinostat, we could rescue the downregulation of *ZMAT3* mRNA upon *Kp*SN as well as Wig-1 and p53 proteins (Fig. 7b, c), confirming HDACs-dependent repression of *ZMAT3*. Altogether, our results demonstrate that activation of the TLR4-NF-κB pathway by bacterial LPS leads to destabilization of *TP53* mRNA through its 3′UTR, mediated by inhibition of RNA-stabilizing factors such as Wig-1.

In conclusion, our data reveal a novel mechanism of p53 inhibition during the cell response to *K. pneumoniae* and other Enterobacteria, through NF-κB-dependent destabilization of *TP53* mRNA, p53 protein downregulation and impairment of its transcriptional activity and tumor suppressive function.

## Discussion

In this study, we discovered that *K. pneumoniae* strongly inhibit p53 and prevent its anti-tumorigenic functions. Interestingly, we found that *K. pneumoniae* LPS was sufficient to inhibit p53. Moreover, our data point out to a more general mechanism of *TP53* mRNA repression by TLR4 agonist LPS from Enterobacteria, which elicits the canonical LPS-TLR4-NF-κB pathway. Our results shed a new perspective on published data about p53 response to bacteria, where inhibition of p53 downstream of microbe-associated molecular patterns (MAMPs) and pattern recognition receptors (PRRs) signaling has not been investigated [15, 42].

**Fig. 8:**
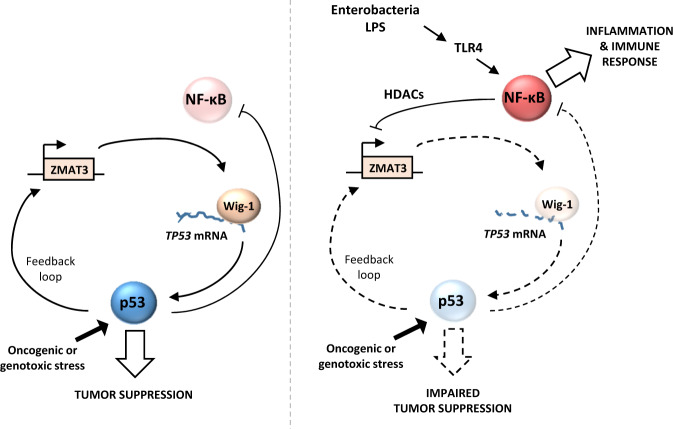
Schematic representation of p53 inhibition by LPS. Upon oncogenic or genotoxic stress (left panel), p53 is stabilized, and induces *ZMAT3*/Wig-1 expression. In turn, Wig-1 stabilizes *TP53* mRNA, leading to a robust p53 accumulation, p53 activity and tumor suppression. Upon LPS signaling (right panel), activation of the canonical TLR4-NF-κB pathway inhibits *ZMAT3* transcription via HDACs, preventing *TP53* mRNA stabilization and ultimately impairing p53 tumor suppressive function. Additionally, p53 is unable to inhibit NF-κB, allowing a robust inflammatory response.

The mechanisms controlling p53 protein stability have been extensively characterized and accepted as the major level of p53 regulation [43]. Only few studies addressed the regulation of *TP53* mRNA during the host response to stress [44–46]. Our findings reveal that *TP53* mRNA serves as an important regulatory platform to turn off p53 activity upon infection and NF-κB activation. The NF-κB and p53 pathways have long been known to be tightly intertwined and to antagonize each other at multiple levels [47, 48]. p53 activation impairs NF-κB function, including in response to LPS [49, 50], and conversely, loss of p53 increases NF-κB activity [51]. Therefore, p53 inhibition in innate immune cells and fibroblasts exposed to MAMPs could be crucial to ensure a proper NF-κB-dependent inflammation and innate immune response. Supporting this hypothesis, p53 KO mice infected by *K. pneumoniae* display an enhanced bacterial clearance by neutrophils and macrophages [52]. Additionally, post-transcriptional mRNA processing have recently emerged as a key level of regulation to coordinate the switch from immune cell proliferation to activation during innate immune response [38]. Our finding that RNA binding factors such as Wig-1 are among the top differentially expressed genes is consistent with this idea. Importantly, Wig-1 forms a positive feedback loop with p53 and it is an important p53 target gene [23, 24]. Thus, our data suggest that *TP53* mRNA destabilization and Wig-1 downregulation cooperate during acute inflammation to robustly activate NF-κB to fight bacterial infection, simultaneously weakening tumor barrier (Fig. 8).

Importantly, CRC patients have elevated circulating LPS and systemic inflammation [53]. Moreover, CRC-associated dysbiosis is usually enriched in Gram-negative bacteria [54], which suggests that LPS secretion is an important factor linking bacterial dysbiosis and tumorigenesis. We found that commensal bacteria expressing a TLR4 antagonist LPS, such as *B. fragilis*, do not downregulate p53, which could contribute to their protective role in colitis-associated colorectal cancer [55]. In addition, colonization of the gut by *Enterococcus faecalis*, a Gram-positive bacterium, induces inflammatory response and colitis similarly to Enterobacteria colonization but does not induce tumorigenesis [56]. Altogether, it suggests that activation of the TLR4-mediated signaling by LPS plays a critical role in promoting cancer not only by inducing inflammation but also by inhibiting p53.

Chronic inflammation linked to bacterial dysbiosis could cause persistent impairment of p53 tumor suppressor function in innate immune cells, which might create a cellular context favorable for oncogenesis. Supporting this idea, mouse models with genetic inactivation of p53 specifically in myeloid cells are more prone to tumorigenesis [57]. Additionally, inactivation of p53 in the tumor microenvironment have profound consequences on the tumor cells themselves in a non-cell autonomous fashion [58, 59]. As tumor progression is associated with disruption of the epithelial layer and invasion of the tumor by bacteria, MAMPs signaling and downstream inhibition of p53 in cancer-associated fibroblasts and tumor-associated macrophages could fuel tumor development. Accordingly, several studies have shown that LPS activation of TLR4 accelerates tumor growth [29, 60]. Interestingly, our analysis of cancer patient data demonstrated an inverse correlation between TLR4 expression and p53 mutation rate in CRC. Our results support the idea that TLR4 activation disables p53 function in the absence of mutation. Moreover, it suggests that the cancer-associated bacterial microbiota exerts a selection pressure on cancer cells and drives tumor evolution. In line with this hypothesis, recent metagenomics analysis of lung cancer microbiota have shown significant differences in bacterial populations associated with p53 wild-type *versus* p53-mutant tumors [61]. Further analyses of metagenomics data from cancer patients in connection with tumor genome profiling are required to understand the connection between the microbiota and host genomic alterations.

## Methods

### Bacterial strains

All bacterial strains used in this study are listed in Supplementary Table S1. *K. pneumoniae* SHG4 and SGH10 strains were a gift from Y.-H. Gan [62, 63], IA565 strain was a gift from G. Huffnagle [64]. Enterobacteria were grown either in DMEM 10% FBS, Luria Broth or low salt Luria Broth at 37 °C. *S. pneumoniae* was grown on agar blood plates at 37 °C with 5% CO_2._ *B. fragilis* was grown on agar blood plates at 37 °C under anaerobic conditions. When stated, 100 μg/mL gentamycin, 50 μg/mL hygromycin, 50 μg/mL apramycin was added to the culture.

*K. pneumoniae lpxM* deletion mutant (∆lpxM) was constructed using a previously established allelic exchange method with a few modifications [65]. Briefly, the apramycin resistance cassette with extensions homologous to regions adjacent to *lpxM* was generated by PCR using the pIJ773 plasmid [65] as a template (primers: forward 5′-CTACACTATCCCATTATCTTGATTAAGCAGTCGATCTGCGGATTGGGCATGATTCCGGGATCGTCGACC-3′; reverse 5′-CGAGTAAGCACGGTAGAGATAAAAAAGCCTCCTGACGGAGGCTTTTTTTATGTAGGCTGGAGCTGCTTC-3′). The PCR product was then electroporated into *K. pneumoniae* cells carrying the pSIM18 plasmid [66]. Replacement of the gene by the apramycin resistance cassette flanked by two Flp recombinase target sequences was confirmed by PCR. The resulting strain was then transformed with the pFLP-hyg plasmid (Addgene #87831; [65]) and incubated for 24 h at 30 °C, allowing excision of the cassette by the Flp recombinase. Plasmid pFLP-hyg was then eliminated at 42 °C, and the cassette excision was verified by sequencing. The pBAD33-gent plasmid producing lpxM was constructed by standard restriction/ligation cloning by inserting an XbaI-*lpxM*-HindIII fragment (obtained by PCR using the primers: forward 5′-GATCCTCTAGAGGATTGGGCATGGAAACGAAAAAAAAT-3′; reverse 5′-GATCCAAGCTTTTATTTCTTTTTCGTGAACAGCTCTTTGCG-3ʹ) into the XbaI-HindIII digested pBAD33-gent plasmid (Addgene #65098; [67]).

### Human cell lines and primary cells

Human immortalized fibroblasts BJ hTert, BJ hTert shp53, BJ hTert HRasV12^ER-Tam^ and BJ hTert HRasV12^ER-Tam^ shp53 cells were obtained from R. Agami [68]. Human melanoma cell line A375 was from ATCC. Primary human normal fibroblasts (HNF) from dermal biopsies were obtained from A. Falk (Karolinska Institute). Cell authentication was performed by STR profiling analysis at Eurofin Genomics. Mycoplasma contamination was tested monthly using MycoAlert Mycoplasma Detection Kit (Lonza) according to the manufacturer’s instructions. All experiments were performed within 10 passages from frozen stocks.

BJ hTert cells with stable STAT3 knock-down (BJ hTert shSTAT3), inducible p65 knock-down (BJ hTert shp65^TET-ON^) or ZMAT3 overexpression (BJ hTert pLV Wig-1; BJ hTert pLV Wig-1*H88A; BJ hTert pLV Wig-1-Flag) were generated by lentiviral transduction and selected 48 h with 2 μg/mL puromycine. Wig-1-overexpressing lentiviral vector (pLV Wig-1) was purchased from VectorBuilder. Site-directed substitution of Wig-1 Histidine 88 to Alanine and Flag tag insertion were introduced by quick change mutagenesis using pLV Wig-1 as matrix, using the following pairs of mutagenic primers: forward 5ʹ-gcccaggctgcttatcagggtaaaaatcatggtaagaaactccgaaattac-3ʹ and reverse 5ʹ- ccctgataagcagcctgggcttgctgtgcagagttcaaggtgacattgc-3 for H88A mutation; forward 5ʹ-gagatggagaatctgggatatgtaGATTATAAAGATGATGATGATAAAtagacccagctttcttgtacaaagtg-3ʹ and reverse 5ʹ- gctgggtctaTTTATCATCATCATCTTTATAATCtacatatcccagattctccatctcattcctgtaccgctgt-3ʹ for Flag insertion. STAT3 shRNA lentiviral vector (pGIPZ shSTAT3), Tet-inducible p65 shRNA lentiviral vectors (pTRIPZ shp65^TET-ON^) and their respective control vectors were purchased from Dharmacon. shRNA are detailed in Supplementary Table S2.

HRas^V12^ was induced in BJ-hTert HRasV12^ER-Tam^ cells by adding 200 nM of 4-hydroxytamoxifen (4-OHT) (Sigma-Aldrich) to the culture medium. shRNA-mediated knock-down of p65 in BJ hTert shp65^TET-ON^ cells was induced by 3 μg/mL of doxycycline (Sigma-Aldrich) for 48 h.

For experiments on PBMC-derived macrophages, monocytes were isolated from anonymous buffy coats of healthy blood donors (Karolinska University Hospital) using Ficoll gradient and centrifugation. Briefly, blood was diluted in PBS and layered on to Ficoll-Paque (GE Healthcare) and centrifuged at 1,200 rpm for 20 min. The interface layer containing the monocytes was collected and monocytes were further washed twice in PBS. Monocytes were then incubated 2 h for adhesion and unattached cells were washed with PBS. For differentiation into M1 and M2 macrophages, monocytes were cultured in RPMI 1640, 2 mM I-glutamine, 10% FBS, streptomycin/penicillin (Sigma-Aldrich) supplemented with increasing concentration of Granulocyte-Macrophage Colony-Stimulating (Sigma; up to 400 ng/ml) or Macrophage Colony-Stimulating Factor human (Sigma-Aldrich; up to 40 ng/ml) for 7 days.

### Transient infections of BJ hTert cells and bacteria cell free supernatant preparation

For transient bacterial infections, overnight DMEM cultures of *K. pneumoniae* were diluted in fresh medium (DMEM, 10% FBS) and 2.10^5^ BJ hTert cells seeded in 6-wells plate were infected with a multiplicity of infection (m.o.i.) of 0.1 to 10 as indicated. After 2 h, cells were washed 3–6 times and incubated in cell culture medium with 200 µg/mL gentamicin for an extra 6 h.

To prepare bacteria cell free supernatant, Enterobacteria strains were cultivated in DMEM 10% FBS at 37 °C until OD reaches 2. *S. pneumoniae* and *B. fragilis* were grown on Blood agar plate overnight at 37 °C and respectively incubated with 5% Co2 or under anaerobic conditions. Supernatant was obtained by centrifugation at 4000 rpm for 15 min. The supernatant fraction was then passed through a 0.22 µm pore size filter (Millipore). Supernatant fractionation was done using concentrators with 3 kDa and 100 kDa cut-off (Pierce). Heat treatment was performed at 75 °C for 15 min.

### Cell treatments

Unless stated otherwise, *Kp*SN and other bacterial supernatants were used to supplement culture medium to a final concentration of 10% v/v for 8 h (for RT-qPCR) or 10 h (for Western blots). Purified *K. pneumoniae* LPS (Sigma-Aldrich) was used at 100 ng/mL for the same duration. Doxorubicin (Sigma-Aldrich) was used at 0.2 μM. For treatments with TAK242, Nutlin, MG132, AKT1/2 kinase inhibitor, calpain inhibitor III, vorinostat (Sigma-Aldrich) and belinostat (VWR), cells were pretreated 2 h with the inhibitor before co-treatment with *Kp*SN or LPS. For treatment with polymyxin B, medium containing *Kp*SN or LPS was incubated 24 h at 37 °C with 30 μg/mL polymixin B sulfate (Sigma-Aldrich) before addition to the cells. For cycloheximide chase assay, cells were pre-treated 4 h with *Kp*SN or LPS, and then treated with 10 μM cycloheximide (Sigma-Aldrich) for the indicated time.

### RT-qPCR

Total RNA extraction and cDNA synthesis were performed using Aurum total RNA and iScript cDNA synthesis kits (Bio-Rad) according to supplier instructions. Relative mRNA levels were measured by RT-qPCR using SsoAdvanced Universal SYBRGreen SuperMix (Bio-Rad). *GAPDH* and *RPL13A* were used as housekeeping genes. Error bars represent standard deviation from mean of at least three independent experiments. The sequences of RT-qPCR primers used are detailed in Supplementary Table S2.

### Western blots

For detection of human proteins, cells were harvested, washed, and lysed in ice cold RIPA buffer (150 mM NaCl; 5 mM Tris pH 8.0; 1% Triton X-100; 0.5% sodium deoxycholate; 0.1% SDS) supplemented with cOmplete protease inhibitor cocktail (Roche) and PhosSTOP phosphatase inhibitors (Roche). Endogenous proteins were detected by standard Western blot using the following antibodies: IκBα (#9242, Cell Signaling); p21 (#610233, BD Transduction); p53 (sc-126, Santa Cruz Biotechnology); p65, phospho-S536 (#3033, Cell Signaling); p65, total (#8242, Cell Signaling); PIG3 (sc-166664, Santa Cruz Biotechnology); Wig-1 (FJ1, raised against a C-terminal Wig-1 peptide, a kind gift from K. Wiman [69]). β-actin (clone C-4, Merk Millipore) was used as loading control. Densitometric quantifications of the bands were performed using ImageJ software and normalized to β-actin level.

For detection of *K. pneumoniae* lpxM, a polyclonal anti-lpxM antiserum was produced by immunizing rabbits with a synthetic peptide, GEIEPYKRKELFTKKK (Singapore Advanced Biologics).

### LPS quantification

Cell-free supernatants preparations were serially diluted in pyrogen-free water, and reactogenic LPS was detected using the Pierce LAL chromogenic endotoxin quantification kit (LifeTechnologies), according to the manufacturer’s instructions but halving the suggested volumes. Reactogenic LPS units for each sample were normalized to the OD_600_ of the culture. All data are presented as the mean and standard error of the mean (SEM) for at least three sets of biological replicates.

### Luciferase reporter assays

*TP53* promoter-Luc plasmid (pGL2-356bp; Addgene #16292) was a gift from W. El-Deiry [70]. Luc-*TP53* UTRs plasmid (145-pGL3ctrl-3ʹ UTR; Addgene #16292) was a gift from M. Kastan [45]. Each construct was co-transfected with a *Renilla* luciferase-expressing vector in BJ hTert cells using Lipofectamine 3000 (ThermoFisher) according to supplier’s instructions 24 h before treatment. Luciferase expression was measured by RT-qPCR and transfection efficiency was normalized using *Renilla* luciferase expression.

### Measurement of oncogene-induced senescence

BJ hTert HRasV12^ER-Tam^ and BJ hTert HRasV12^ER-Tam^ shp53 cells were seeded in 12-wells plates on glass coverslips, then treated the following day with 4-OHT and *Kp*SN. The culture medium was renewed every 3 days, and *Kp*SN was added each day to the culture medium. Senescence was measured 8 days after the start of the experiments. For β-galactosidase assay, cells were fixed and stained 24 h using the Senescence Cells Histochemical Staining kit (Sigma-Aldrich) according to manufacturer’s instructions. For EdU incorporation Assay, 10 μM EdU was added to the culture medium 24 h before fixation, then EdU was labeled using the Click-iT EdU Alexa Fluor 488 Imaging Kit (Thermo Fisher Scientific). For both assay, quantification was performed by counting positive cells in n ⩾ 200 cells in three independent experiments.

### RIP-qPCR

RNA immunoprecipitation (RIP) was performed on 2.10^7^ BJ hTert pLV Wig-1-Flag cells using the Magna RIP Kit (Merck-Millipore) according to the supplier instructions. Wig-1-Flag immunoprecipitation was performed using a monoclonal anti-Flag antibody (F3165, Sigma-Aldrich) and mouse IgG as control. Isolated RNA was then analyzed by RT-qPCR.

### RNA sequencing

BJ hTert and BJ hTert shp53 were treated for 8 h with *Kp*SN and 0.2 μM doxorubicin in three independent biological replicates. Total RNA was extracted using the *mir*Vana miRNA isolation kit (Ambion). RNA-seq was performed by Vertis Biotechnologie AG. Random-primed cDNA libraries were prepared according to Illumina protocol. The cDNA pool was paired-end sequenced on an Illumina NextSeq 500 system using 2 × 150 bp read length. Quality control was performed using FastQC. nf-core/rnaseq pipeline (v1.4) was used to process the reads [71]. HISAT2 (v2.1.0) was used to align the raw RNA-seq fastq reads to the human reference genome (GRCh38.97). Read quantification was computed using featureCounts (v1.6.4). Differential analysis was performed with the R package DESeq2. False positive discovery (FDR) < 0.05 was set as cut-off for differentially expressed genes (DEGs). DEGs were plotted using R package ggplot2. Gene ontology (GO: biological process) and pathway enrichment analysis was performed using g:Profiler [72] and visualized using Cytoscape (v3.6.1) [73] as described by Reimand *et al*. [74]. The complete lists of differentially expressed genes and enriched GO terms upon *Kp*SN treatment are provided in Supplementary Table S3. To define a high confidence gene set of p53 targets in BJ hTert cells, we intersected genes upregulated upon doxorubicin treatment, repressed upon p53 shRNA and p53 core targets genes defined by Fisher *et al*. [22]. Complete list of the 161 high confidence p53 target genes is provided in Supplementary Table S4.

### Public database patient data analysis

Patient data from The Cancer Genome Atlas (TCGA; https://www.cancer.gov/tcga) were analyzed using cBioPortal for Cancer Genomics (http://cbioportal.org) [75, 76]. Normalized expression data and *TP53* mutations from early stage (grade I-II) Colorectal adenocarcinoma (COAD), Breast carcinoma (BRCA), Liver hepatocellular carcinoma (LIHC) and Lung adenocarcinoma (LUAD) from TCGA PanCancer Atlas Studies were analyzed. Tumors were classified based on *TLR4* expression Z-score across all samples as high *TLR4* (Z-score > 0.75) or low *TLR4* (Z-score < −0.75).

### Statistical analyses

Unless otherwise stated, statistical significance was calculated using two-tailed Student t-test from at least three independent experiments.

## Supplementary information

Supplementary Figures

Supplementary Tables S1 and S2

Supplementary Table S3

Supplementary Table S4

## Data Availability

The datasets generated in the study are available in GEO database (https://www.ncbi.nlm.nih.gov/geo/) under accession number GSE174531.

## References

[1] de Martel C, Ferlay J, Franceschi S, Vignat J, Bray F, Forman D (2012). Global burden of cancers attributable to infections in 2008: a review and synthetic analysis. Lancet Oncol.

[2] Sepich-Poore GD, Zitvogel L, Straussman R, Hasty J, Wargo JA, Knight R. The microbiome and human cancer. Science. 2021;371. 10.1126/science.abc4552 PMC8767999 33766858

[3] Wirbel J, Pyl PT, Kartal E, Zych K, Kashani A, Milanese A (2019). Meta-analysis of fecal metagenomes reveals global microbial signatures that are specific for colorectal cancer. Nat Med.

[4] Mei Q-X, Huang C-L, Luo S-Z, Zhang X-M, Zeng Y, Lu Y-Y (2018). Characterization of the duodenal bacterial microbiota in patients with pancreatic head cancer vs. healthy controls. Pancreatology.

[5] Watanabe T, Tada M, Nagai H, Sasaki S, Nakao M (1998). Helicobacter pylori infection induces gastric cancer in mongolian gerbils. Gastroenterology.

[6] Ge Z, Rogers AB, Feng Y, Lee A, Xu S, Taylor NS (2007). Bacterial cytolethal distending toxin promotes the development of dysplasia in a model of microbially induced hepatocarcinogenesis. Cell Microbiol.

[7] Cuevas-Ramos G, Petit CR, Marcq I, Boury M, Oswald E, Nougayrède J-P (2010). Escherichia coli induces DNA damage in vivo and triggers genomic instability in mammalian cells. Proc Natl Acad Sci USA.

[8] Brennan CA, Garrett WS (2019). Fusobacterium nucleatum - symbiont, opportunist and oncobacterium. Nat Rev Microbiol.

[9] Hafner A, Bulyk ML, Jambhekar A, Lahav G (2019). The multiple mechanisms that regulate p53 activity and cell fate. Nat Rev Mol Cell Biol.

[10] Rivas C, Aaronson SA, Munoz-Fontela C (2010). Dual Role of p53 in Innate Antiviral Immunity. Viruses.

[11] Tornesello ML, Annunziata C, Tornesello AL, Buonaguro L, Buonaguro FM. Human oncoviruses and p53 tumor suppressor pathway deregulation at the origin of human cancers. Cancers. 2018;10. 10.3390/cancers10070213 PMC6071257 29932446

[12] Wei J, Nagy TA, Vilgelm A, Zaika E, Ogden SR, Romero-Gallo J (2010). Regulation of p53 tumor suppressor by Helicobacter pylori in gastric epithelial cells. Gastroenterology.

[13] Horvat A, Noto JM, Ramatchandirin B, Zaika E, Palrasu M, Wei J (2018). Helicobacter pylori pathogen regulates p14ARF tumor suppressor and autophagy in gastric epithelial cells. Oncogene.

[14] Buti L, Spooner E, Van der Veen AG, Rappuoli R, Covacci A, Ploegh HL (2011). Helicobacter pylori cytotoxin-associated gene A (CagA) subverts the apoptosis-stimulating protein of p53 (ASPP2) tumor suppressor pathway of the host. Proc Natl Acad Sci USA.

[15] González E, Rother M, Kerr MC, Al-Zeer MA, Abu-Lubad M, Kessler M (2014). Chlamydia infection depends on a functional MDM2-p53 axis. Nat Commun.

[16] Zella D, Curreli S, Benedetti F, Krishnan S, Cocchi F, Latinovic OS (2018). Mycoplasma promotes malignant transformation in vivo, and its DnaK, a bacterial chaperone protein, has broad oncogenic properties. Proc Natl Acad Sci USA.

[17] Wang T, Cai G, Qiu Y, Fei N, Zhang M, Pang X (2012). Structural segregation of gut microbiota between colorectal cancer patients and healthy volunteers. ISME J.

[18] Huang W-K, Chang JW-C, See L-C, Tu H-T, Chen J-S, Liaw C-C (2012). Higher rate of colorectal cancer among patients with pyogenic liver abscess with Klebsiella pneumoniae than those without: an 11-year follow-up study. Colorectal Dis.

[19] Jeong SW, Jang JY, Lee TH, Kim HG, Hong SW, Park SH (2012). Cryptogenic pyogenic liver abscess as the herald of colon cancer. J Gastroenterol Hepatol.

[20] Lan Y, Zhou M, Jian Z, Yan Q, Wang S, Liu W (2019). Prevalence of pks gene cluster and characteristics of Klebsiella pneumoniae-induced bloodstream infections. J Clin Lab Anal.

[21] Kastenhuber ER, Lowe SW (2017). Putting p53 in context. Cell.

[22] Fischer M, Grossmann P, Padi M, DeCaprio JA (2016). Integration of TP53, DREAM, MMB-FOXM1 and RB-E2F target gene analyses identifies cell cycle gene regulatory networks. Nucleic Acids Res.

[23] Janic A, Valente LJ, Wakefield MJ, Di Stefano L, Milla L, Wilcox S (2018). DNA repair processes are critical mediators of p53-dependent tumor suppression. Nat Med.

[24] Bieging-Rolett KT, Kaiser AM, Morgens DW, Boutelle AM, Seoane JA, Van Nostrand EL (2020). Zmat3 is a key splicing regulator in the p53 tumor suppression program. Mol Cell.

[25] Ferbeyre G, de Stanchina E, Lin AW, Querido E, McCurrach ME, Hannon GJ (2002). Oncogenic ras and p53 cooperate to induce cellular senescence. Mol Cell Biol.

[26] Berezow AB, Ernst RK, Coats SR, Braham PH, Karimi-Naser LM, Darveau RP (2009). The structurally similar, penta-acylated lipopolysaccharides of Porphyromonas gingivalis and Bacteroides elicit strikingly different innate immune responses. Micro Pathog.

[27] Menendez D, Shatz M, Azzam K, Garantziotis S, Fessler MB, Resnick MA (2011). The toll-like receptor gene family is integrated into human DNA damage and p53 networks. PLoS Genet.

[28] Hilty M, Burke C, Pedro H, Cardenas P, Bush A, Bossley C (2010). Disordered microbial communities in asthmatic airways. PloS One.

[29] Dapito DH, Mencin A, Gwak G-Y, Pradere J-P, Jang M-K, Mederacke I (2012). Promotion of hepatocellular carcinoma by the intestinal microbiota and TLR4. Cancer Cell.

[30] Siegl C, Rudel T (2015). Modulation of p53 during bacterial infections. Nat Rev Microbiol.

[31] Bergounioux J, Elisee R, Prunier A-L, Donnadieu F, Sperandio B, Sansonetti P (2012). Calpain activation by the Shigella flexneri effector VirA regulates key steps in the formation and life of the bacterium’s epithelial niche. Cell Host Microbe.

[32] Niu G, Wright KL, Ma Y, Wright GM, Huang M, Irby R (2005). Role of Stat3 in regulating p53 expression and function. Mol Cell Biol.

[33] Joruiz SM, Bourdon J-C p53 isoforms: key regulators of the cell fate decision. Cold Spring Harb Perspect Med. 2016;6. 10.1101/cshperspect.a026039 PMC4968168 26801896

[34] Haronikova L, Olivares-Illana V, Wang L, Karakostis K, Chen S, Fåhraeus R (2019). The p53 mRNA: an integral part of the cellular stress response. Nucleic Acids Res.

[35] Vilborg A, Glahder JA, Wilhelm MT, Bersani C, Corcoran M, Mahmoudi S (2009). The p53 target Wig-1 regulates p53 mRNA stability through an AU-rich element. Proc Natl Acad Sci USA.

[36] Burns DM, Richter JD (2008). CPEB regulation of human cellular senescence, energy metabolism, and p53 mRNA translation. Genes Dev.

[37] Díaz-Muñoz MD, Kiselev VY, Le Novère N, Curk T, Ule J, Turner M (2017). Tia1 dependent regulation of mRNA subcellular location and translation controls p53 expression in B cells. Nat Commun.

[38] Pai AA, Baharian G, Pagé Sabourin A, Brinkworth JF, Nédélec Y, Foley JW (2016). Widespread Shortening of 3’ Untranslated Regions and Increased Exon Inclusion Are Evolutionarily Conserved Features of Innate Immune Responses to Infection. PLoS Genet.

[39] Bersani C, Huss M, Giacomello S, Xu L-D, Bianchi J, Eriksson S (2016). Genome-wide identification of Wig-1 mRNA targets by RIP-Seq analysis. Oncotarget.

[40] Bersani C, Xu L-D, Vilborg A, Lui W-O, Wiman KG (2014). Wig-1 regulates cell cycle arrest and cell death through the p53 targets FAS and 14-3-3σ. Oncogene.

[41] Nguyen HCB, Adlanmerini M, Hauck AK, Lazar MA (2020). Dichotomous engagement of HDAC3 activity governs inflammatory responses. Nature.

[42] Costa L, Corre S, Michel V, Le Luel K, Fernandes J, Ziveri J (2020). USF1 defect drives p53 degradation during Helicobacter pylori infection and accelerates gastric carcinogenesis. Gut.

[43] Levine AJ (2020). p53: 800 million years of evolution and 40 years of discovery. Nat Rev Cancer.

[44] Fu L, Minden MD, Benchimol S (1996). Translational regulation of human p53 gene expression. EMBO J.

[45] Chen J, Kastan MB (2010). 5′-3′-UTR interactions regulate p53 mRNA translation and provide a target for modulating p53 induction after DNA damage. Genes Dev.

[46] Gajjar M, Candeias MM, Malbert-Colas L, Mazars A, Fujita J, Olivares-Illana V (2012). The p53 mRNA-Mdm2 interaction controls Mdm2 nuclear trafficking and is required for p53 activation following DNA damage. Cancer Cell.

[47] Ak P, Levine AJ (2010). p53 and NF-κB: different strategies for responding to stress lead to a functional antagonism. FASEB J.

[48] Gudkov AV, Komarova EA p53 and the carcinogenicity of chronic inflammation. Cold Spring Harb Perspect Med. 2016;6. 10.1101/cshperspect.a026161 PMC5088512 27549311

[49] Meylan E, Dooley AL, Feldser DM, Shen L, Turk E, Ouyang C (2009). Requirement for NF-kappaB signalling in a mouse model of lung adenocarcinoma. Nature.

[50] Liu G, Park Y-J, Tsuruta Y, Lorne E, Abraham E (2009). p53 Attenuates lipopolysaccharide-induced NF-kappaB activation and acute lung injury. J Immunol.

[51] Komarova EA, Krivokrysenko V, Wang K, Neznanov N, Chernov MV, Komarov PG (2005). p53 is a suppressor of inflammatory response in mice. FASEB J.

[52] Madenspacher JH, Azzam KM, Gowdy KM, Malcolm KC, Nick JA, Dixon D (2013). p53 Integrates host defense and cell fate during bacterial pneumonia. J Exp Med.

[53] de Waal GM, de Villiers WJS, Forgan T, Roberts T, Pretorius E (2020). Colorectal cancer is associated with increased circulating lipopolysaccharide, inflammation and hypercoagulability. Sci Rep..

[54] Wu N, Yang X, Zhang R, Li J, Xiao X, Hu Y (2013). Dysbiosis signature of fecal microbiota in colorectal cancer patients. Micro Ecol.

[55] Lee YK, Mehrabian P, Boyajian S, Wu W-L, Selicha J, Vonderfecht S, et al. The protective role of bacteroides fragilis in a murine model of colitis-associated colorectal cancer. mSphere. 2018;3. 10.1128/mSphere.00587-18 PMC6236802 30429227

[56] Arthur JC, Perez-Chanona E, Mühlbauer M, Tomkovich S, Uronis JM, Fan T-J (2012). Intestinal inflammation targets cancer-inducing activity of the microbiota. Science.

[57] He X-Y, Xiang C, Zhang C-X, Xie Y-Y, Chen L, Zhang G-X (2015). p53 in the myeloid lineage modulates an inflammatory microenvironment limiting initiation and invasion of intestinal tumors. Cell Rep..

[58] Procopio M-G, Laszlo C, Al Labban D, Kim DE, Bordignon P, Jo S-H (2015). Combined CSL and p53 downregulation promotes cancer-associated fibroblast activation. Nat Cell Biol.

[59] Arandkar S, Furth N, Elisha Y, Nataraj NB, van der Kuip H, Yarden Y (2018). Altered p53 functionality in cancer-associated fibroblasts contributes to their cancer-supporting features. Proc Natl Acad Sci USA.

[60] Fukata M, Chen A, Vamadevan AS, Cohen J, Breglio K, Krishnareddy S (2007). Toll-like receptor-4 promotes the development of colitis-associated colorectal tumors. Gastroenterology.

[61] Greathouse KL, White JR, Vargas AJ, Bliskovsky VV, Beck JA, von Muhlinen N (2018). Interaction between the microbiome and TP53 in human lung cancer. Genome Biol.

[62] Lam MMC, Wyres KL, Duchêne S, Wick RR, Judd LM, Gan Y-H (2018). Population genomics of hypervirulent Klebsiella pneumoniae clonal-group 23 reveals early emergence and rapid global dissemination. Nat Commun.

[63] Lee IR, Molton JS, Wyres KL, Gorrie C, Wong J, Hoh CH (2016). Differential host susceptibility and bacterial virulence factors driving Klebsiella liver abscess in an ethnically diverse population. Sci Rep..

[64] Hornick DB, Thommandru J, Smits W, Clegg S (1995). Adherence properties of an mrkD-negative mutant of Klebsiella pneumoniae. Infect Immun.

[65] Huang T-W, Lam I, Chang H-Y, Tsai S-F, Palsson BO, Charusanti P (2014). Capsule deletion via a λ-Red knockout system perturbs biofilm formation and fimbriae expression in Klebsiella pneumoniae MGH 78578. BMC Res Notes.

[66] Chan W, Costantino N, Li R, Lee SC, Su Q, Melvin D (2007). A recombineering based approach for high-throughput conditional knockout targeting vector construction. Nucleic Acids Res.

[67] Jimenez N, Lacasta A, Vilches S, Reyes M, Vazquez J, Aquillini E (2009). Genetics and proteomics of Aeromonas salmonicida lipopolysaccharide core biosynthesis. J Bacteriol.

[68] Kolfschoten IGM, van Leeuwen B, Berns K, Mullenders J, Beijersbergen RL, Bernards R (2005). A genetic screen identifies PITX1 as a suppressor of RAS activity and tumorigenicity. Cell.

[69] Zhang M, Heldin A, Palomar-Siles M, Öhlin S, Bykov VJN, Wiman KG (2017). Synergistic rescue of nonsense mutant tumor suppressor p53 by combination treatment with aminoglycosides and Mdm2 inhibitors. Front Oncol.

[70] Wang S, El-Deiry WS (2006). p73 or p53 directly regulates human p53 transcription to maintain cell cycle checkpoints. Cancer Res.

[71] Ewels PA, Peltzer A, Fillinger S, Patel H, Alneberg J, Wilm A (2020). The nf-core framework for community-curated bioinformatics pipelines. Nat Biotechnol.

[72] Reimand J, Arak T, Adler P, Kolberg L, Reisberg S, Peterson H (2016). g:Profiler-a web server for functional interpretation of gene lists (2016 update). Nucleic Acids Res.

[73] Shannon P, Markiel A, Ozier O, Baliga NS, Wang JT, Ramage D (2003). Cytoscape: a software environment for integrated models of biomolecular interaction networks. Genome Res.

[74] Reimand J, Isserlin R, Voisin V, Kucera M, Tannus-Lopes C, Rostamianfar A (2019). Pathway enrichment analysis and visualization of omics data using g:Profiler, GSEA, Cytoscape and EnrichmentMap. Nat Protoc.

[75] Cerami E, Gao J, Dogrusoz U, Gross BE, Sumer SO, Aksoy BA (2012). The cBio cancer genomics portal: an open platform for exploring multidimensional cancer genomics data. Cancer Disco.

[76] Gao J, Aksoy BA, Dogrusoz U, Dresdner G, Gross B, Sumer SO (2013). Integrative analysis of complex cancer genomics and clinical profiles using the cBioPortal. Sci Signal.

