# Supplementary material for: Enterobacteria impair host p53 tumor suppressor activity through mRNA destabilization
Source: Oncogene. 2022 Feb 23;41(15):2173–86. doi: 10.1038/s41388-022-02238-5 (PMC8993692; doi:10.1038/s41388-022-02238-5)
Supplement: Supplementary file 1 — Supplementary Figures [file 41388_2022_2238_MOESM1_ESM.pdf]

SUPPLEMENTARY FIGURE S1

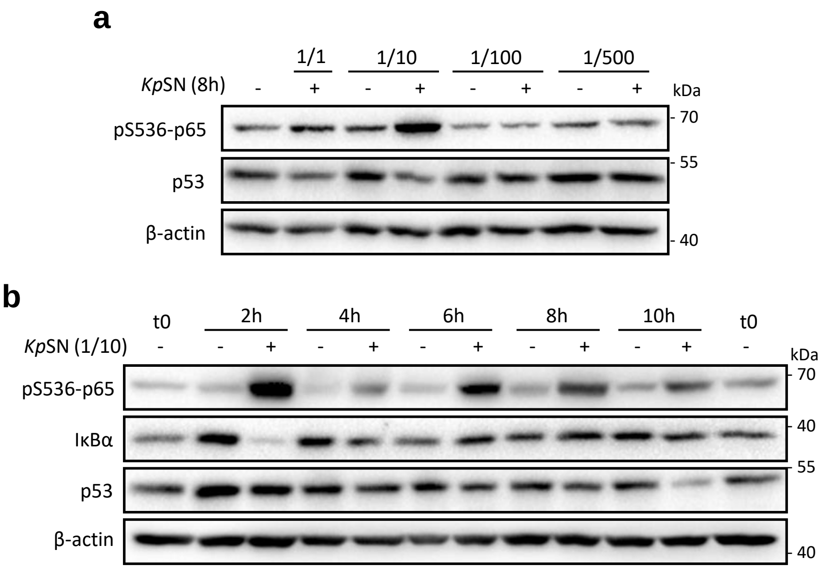

Supplementary Figure S1. a, Western blot for dose-response of BJ hTert cells to different dilutions of *Kp*SN in cell culture medium. Negative control for pure *Kp*SN was normal cell culture medium. For other dilutions, water supplementation of cell culture medium instead of *Kp*SN was used as negative control. **b,** Western blot for time course response of BJ hTert cells to *Kp*SN.

SUPPLEMENTARY FIGURE S2

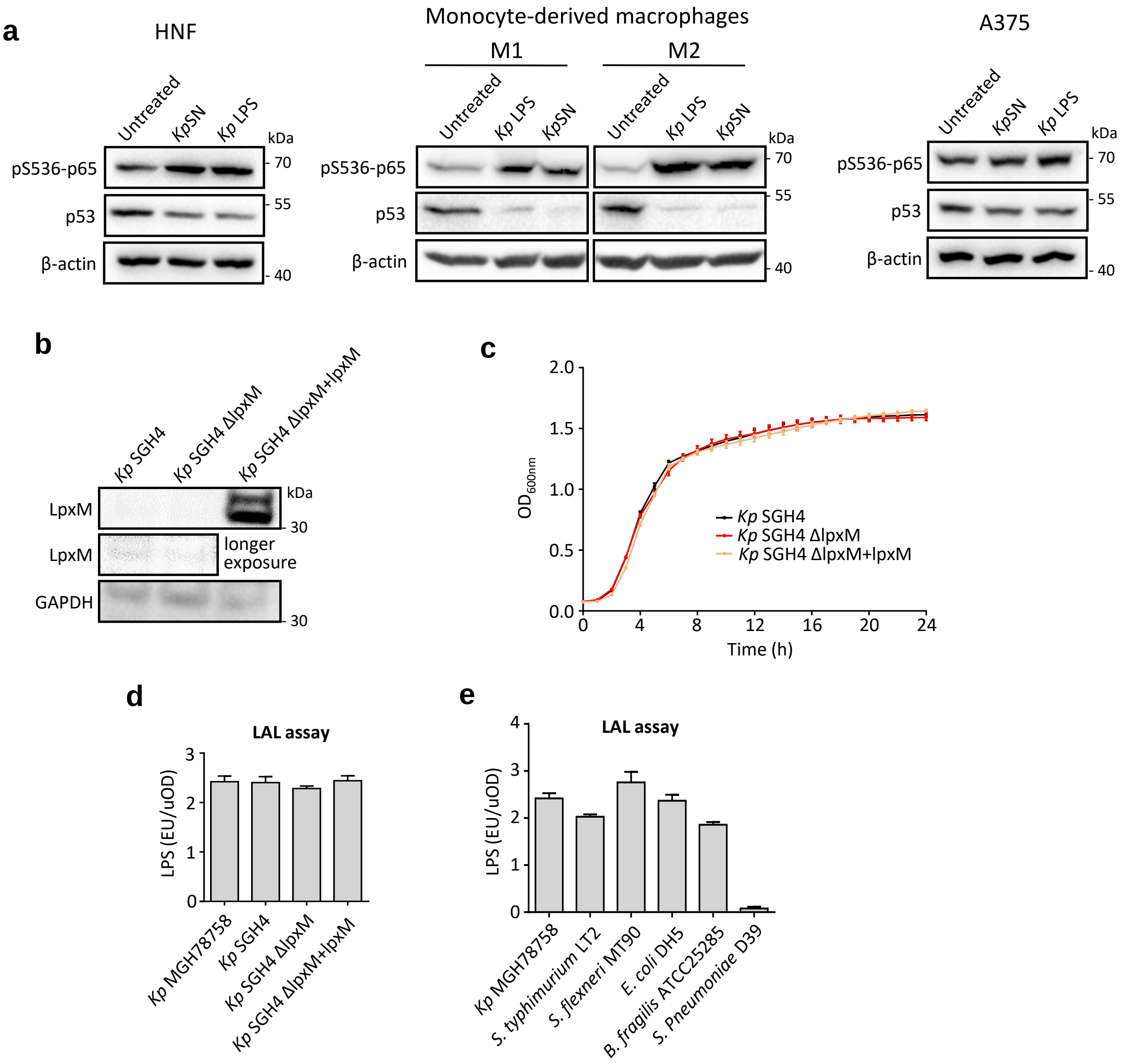

Supplementary Figure S2. a, Western blot of human normal primary fibroblasts (HNF), PBMC-derived macrophages polarized in M1 or M2 phenotype and A375 melanoma cell line upon 10 h exposure to *Kp*SN or LPS (100 ng/mL). **b**, Western blot for *lpxm* deletion (*ΔlpxM*) and complementation (*ΔlpxM+lpxM*) in *K. pneumoniae* SGH4 strain. **c**, Growth curve of *K. pneumoniae ΔlpxM* and *ΔlpxM+lpxM* compared to parental SGH4 strain. **d**, Relative LPS quantification by LAL assay of supernatant from *K. pneumoniae ΔlpxM* and *ΔlpxM+lpxM* compared to parental SGH4 strain. **e**, Relative LPS quantification by LAL assay of the supernatant from different bacteria used in Fig. 4g.

SUPPLEMENTARY FIGURE S3

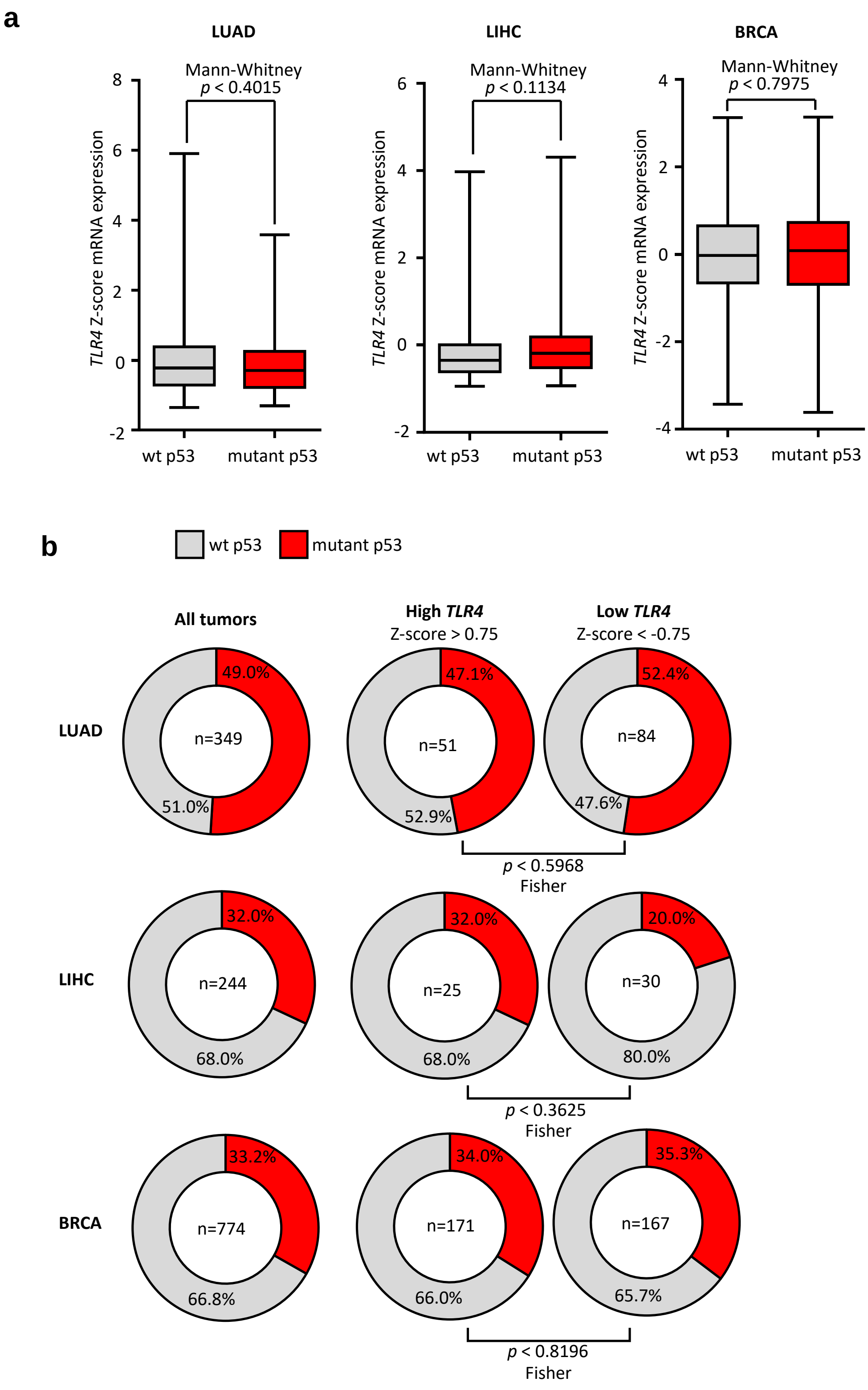

Supplementary Figure S3. a, Absence of correlation between TLR4 expression and p53 mutation in early grade lung adenocarcinoma (LUAD), liver hepatocarcinoma (LIHC) and breast carcinoma (BRCA) patients data from TCGA. **b**, Comparison of p53 mutation rate between tumors with low and high TLR4 expression for early grade LUAD, LIHC and BRCA.

SUPPLEMENTARY FIGURE S4

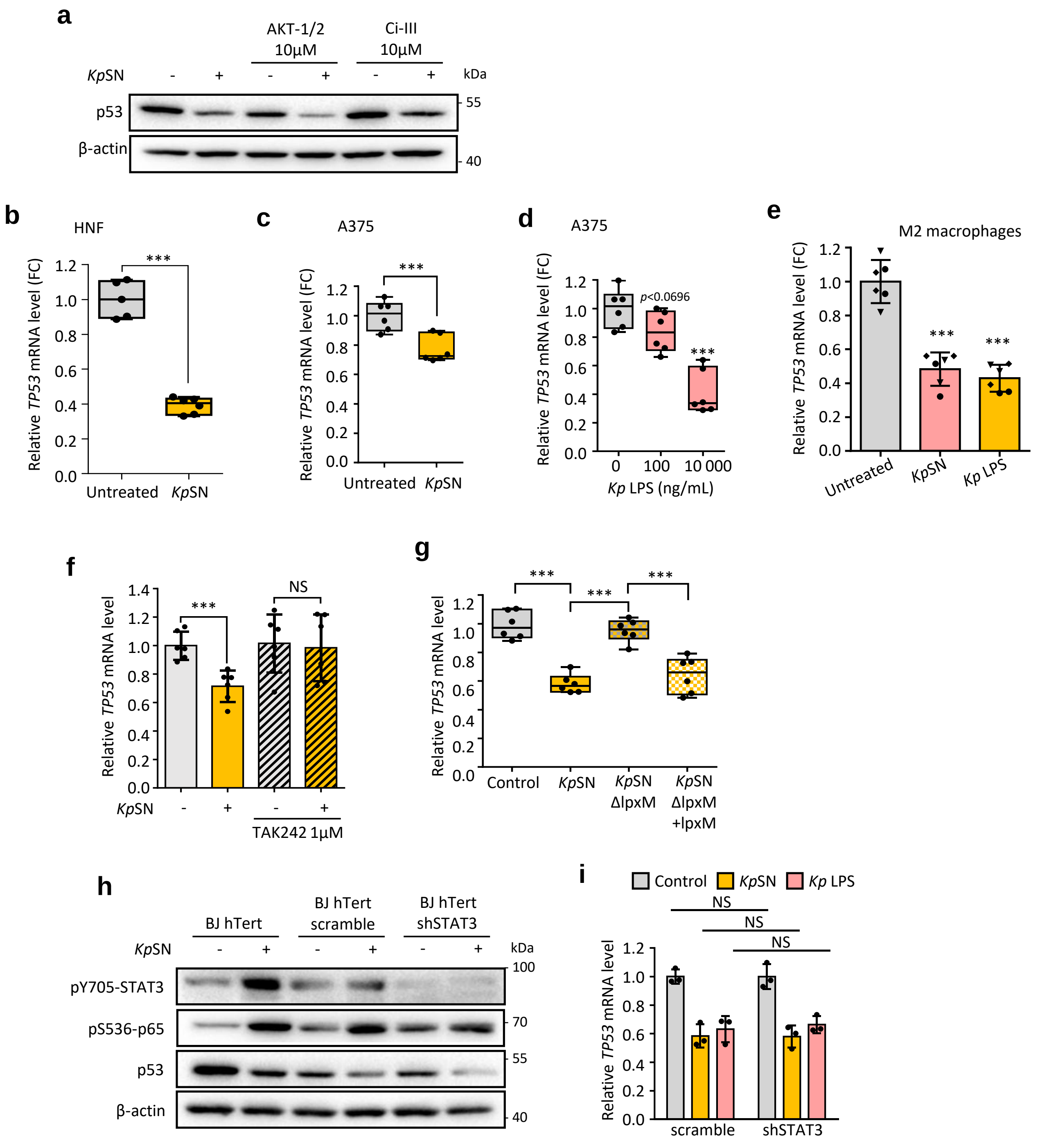

Supplementary Figure S4. a, Downregulation of p53 protein level induced by *Kp*SN in BJ hTert cells is not rescued by AKT inhibitor (AKT-1/2) or calpain inhibitor (Ci-III). **b**, RT-qPCR for *TP53* mRNA level upon *Kp*SN treatment of human normal primary fibroblasts (HNF). **c**, RT-qPCR for *TP53* mRNA level upon *Kp*SN treatment of A375 melanoma cell line. **d**, RT-qPCR for *TP53* mRNA level upon LPS treatment of A375 melanoma cell line. **e**, RT-qPCR for *TP53* mRNA level upon *Kp*SN or 100 ng/mL LPS (8 h) in PBMC-derived macrophages, polarized into M2 phenotype. The different data point symbols indicate different blood donors. **f**, TLR4 dependency of *Kp*SN-induced *TP53* mRNA downregulation assessed by RT-qPCR upon treatment with TLR4 inhibitor TAK242 in BJ hTert cells. **g**, RT-qPCT for *TP53* mRNA level upon exposure to supernatant from *K. pneumoniae ΔlpxM* and *ΔlpxM+lpxM*. **h**, STAT3 independency of *Kp*SN-induced p53 downregulation assessed by Western blots using stable STAT3 shRNA-mediated knock-down BJ hTert cells. **i**, RT-qPCR for *TP53* mRNA level in BJ hTert cells with stable STAT3 shRNA-mediated knock-down upon 8 h *Kp*SN or LPS (100ng/mL). * $p<0.05$; *** $p<0.01$; NS non-significant.

SUPPLEMENTARY FIGURE S5

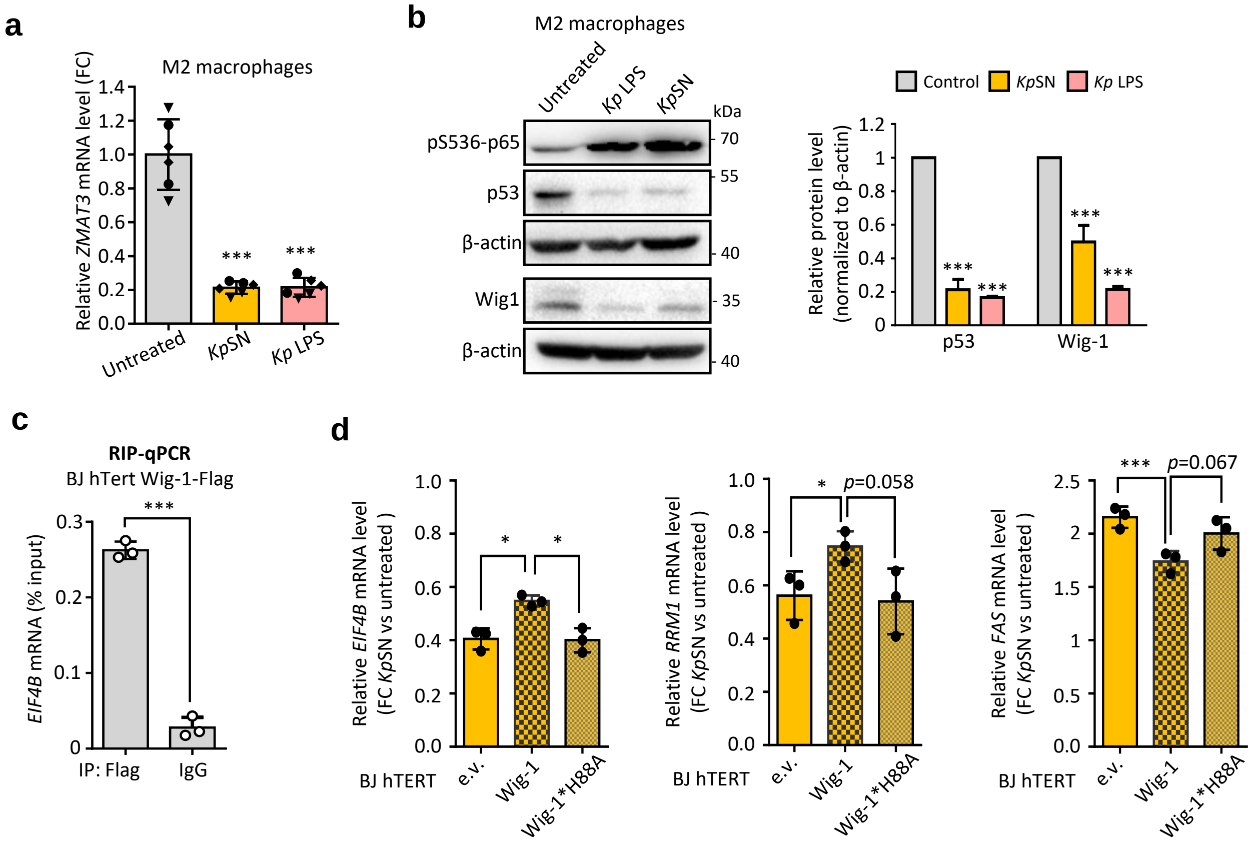

Supplementary Figure S5. a, RT-qPCR for *ZMAT3* mRNA level upon *Kp*SN or 100 ng/mL LPS (8 h) in PBMC-derived macrophages, polarized into M2 phenotype. The different data point symbols indicate different blood donors. *** $p<0.01$. **b**, Left panel: Western blot of M2 macrophages upon *Kp*SN or LPS treatment. Right panel: densitometric quantification of p53 and Wig-1 Western blot bands normalized to β-actin level. **c**, Validation of Wig-1 binding to *EIF4B* mRNA in BJ hTert cells assessed by RIP-qPCR. **d**, RT-qPCR for Wig-1 targets *EIF4B*, *RRM1* and *FAS* upon overexpression of wild type or RNA-binding deficient H88A mutant Wig-1 show that Wig-1 downregulation by *Kp*SN participates in *EIF4B* and *RRM1* repression and *FAS* activation.
