# Supplementary material for: Enterobacteria impair host p53 tumor suppressor activity through mRNA destabilization
Source: Oncogene. 2022 Feb 23;41(15):2173–86. doi: 10.1038/s41388-022-02238-5 (PMC8993692; doi:10.1038/s41388-022-02238-5)
Supplement: Supplementary file 2 — Supplementary Tables S1 and S2 [file 41388_2022_2238_MOESM2_ESM.pdf]

Species	Strain	Source
Klebsiella pneumoniae	MGH78578	ATCC
Klebsiella pneumoniae	SGH10	Lam *et al.* (Ref.[62])
Klebsiella pneumoniae	SGH4	Lee *et al.* (Ref.[63])
Klebsiella pneumoniae	NCTC204	Lab collection
Klebsiella pneumoniae	NCTC5056	Lab collection
Klebsiella pneumoniae	IA565	Hornick *et al.* (Ref.[64])
Klebsiella pneumoniae	SGH4 ΔlpxM	This study
Salmonella enterica serovar *Typhimurium*	SR-11	Lab collection
Shigella flexineri	MT90	Lab collection
Bacterioides fragilis	ATCC 25285	Lab collection
Streptococcus pneumoniae	D39	Lab collection
Escherichia coli	DH5α	Lab collection

Supplementary Table S1. Bacterial strains.

**Sequences of RT-qPCR primers**		
Gene	Forward primer sequence (5’ to 3’)	Reverse primer sequence (5’ to 3’)
CDKN1A (p21)	CTGTCACTGTCTTGTACCC	AGTGGTAGAAATCTGTCATGC
EIF4B	TACAGACTGGAGGGCTCGTC	CCGATACCGGTCTGAATCAT
FAS	GTGACCCTTGCACCAAATGT	GACAAAGCCACCCCAAGTTA
PMAIP1 (NOXA)	CATGAGGGGACTCCTTCAAA	TTCCATCTTCCGTTTCCAAG
RRM1	CTGCTGGAGGAATTGGTGTT	CTCTCAGCATCGGTACAAGG
SESN1	ATTCGGCTGTGGAATCAGTC	TCCACACTGTGATTGCCATT
TP53	TTTGGGTCTTTGAACCCTTG	CCACAACAAAACACCAGTGC
TP53, P1 isoforms	CCCCTGTCATCTTCTGTCCC	ACATCTTGTTGAGGGCAGGG
TP53, P2 isoforms	TAGACGCCAACTCTCTCTAG	AGTCAGGGCACAAGTGAACA
TP53I3 (PIG3)	TAGCCGTGCACTTTGACAAG	ACTGGCCTTGTCTCTGCATT
TP53INP1	CAGAAATAGCCTTCGTCGCC	GTAATTGTACTGACGCGGGC
ZMAT3 (Wig-1)	GAATGAGCAATGTGGTCGAG	GGAACTGAAGGAGGCATCAC
rLuc (*Renilla* luciferase)	AACGCGGCCTCTTCTTATTT	ACCAGATTTGCCTGATTTGC
fLuc (*Firefly* luciferase)	TTCCATCTTCCAGGGATACG	ATCCAGATCCACAACCTTCG
**Sequences of shRNAs**		
Gene	Sequence of mature antisense (5’ to 3’)	
RELA (p65)	TCTATAGGAACTTGGAAGG	
STAT3	ATTGCTGCAGGTCGTTGGT	

Supplementary Table S2. RT-qPCR primers and shRNAs.
